# Calcium phosphate nanoparticles-based systems for siRNA delivery

**DOI:** 10.1093/rb/rbw010

**Published:** 2016-03-04

**Authors:** Xiaochun Xu, Zehao Li, Xueqin Zhao, Lawrence Keen, Xiangdong Kong

**Affiliations:** ^1^Institute of Biomaterials and Marine Biological Resources, College of Life Sciences, Zhejiang Sci-Tech University, Hangzhou 310018, China;; ^2^College of Materials and Textiles, Zhejiang Sci-Tech University, Hangzhou 310018, China

**Keywords:** calcium phosphate, siRNA, nanoparticles, delivery

## Abstract

Despite the enormous therapeutic potential of siRNA as a treatment strategy, the delivery is still a problem due to unfavorable biodistribution profiles and poor intracellular bioavailability. Calcium phosphate (CaP) co-precipitate has been used for nearly 40 years for *in vitro* transfection due to its non-toxic nature and simplicity of preparation. The surface charge of CaP will be tuned into positive by surface modification, which is important for siRNA loading and crossing cell membrane without enzymatic degradation. The new siRNA carrier system will also promote the siRNA escape from lysosome to achieve siRNA sustained delivery and high-efficiency silence. In this review, we focus on the current research activity in the development of CaP nanoparticles for siRNA delivery. These nanoparticles are mainly classified into lipid coated, polymer coated and various other types for discussion.

## Introduction

siRNA (small interfering RNA) is a short dsRNA (typically 20–27 bp), which is known for its ability to silence gene expression in a sequence specific manner (RNA interference) [1, 2]. siRNA is produced when long double-stranded RNA precursors are cleaved by the endonuclease dicer into smaller molecules, and then enter the RNA-induced silencing complex [3–6]. RNA interference was investigated for therapeutic applications after it was discovered in the 1990s [7]. If a disease-related gene could be silenced by RNA interference, then symptoms would be eliminated, therefore siRNA offers a new type to treat a wide variety of diseases [8–10]. However, the utilization of RNA interference in medical treatment currently has found a challenge in siRNA intracellular delivery. This is because of the polyanionic nature of siRNA that prevents efficient diffusion through cellular membranes [11, 12]. Moreover, free siRNA shows unfavorable pharmacokinetic profiles due to their rapid blood degradation and elimination within the cells or systems [13]. A feasible method of siRNA delivery is to look for an ideal carrier, which is safe, inexpensive as well as being easy to produce, and should protect siRNA from either premature enzymatic degradation by avoiding opsonization and subsequent rapid clearance. The siRNA-loading carrier should be taken up by the target cell where it can release its cargo accurately [14, 15].

Various materials, most typically lipids, polymers and partial inorganic nanomaterials have been utilized in siRNA delivery system. The lipids are logical choice in siRNA delivery systems because the cell membrane is rich in lipids [16–18]. As a typical representative, polyethylenimine (PEI) has been commonly used as the standard for siRNA delivery because of its high cellular uptake and efficient endosomal escape [19–22]. However, lipids and polymers both have been found to have a toxic effect in cell lines [23, 24]. It was shown that inflammatory toxicity and liver toxicity occurred after systemic administration of lipid nanocarriers to mice [25, 26]. The toxicity of PEI partly comes from its limited biodegradability. When PEI molecules are released from polyplexes, free PEI molecules will interact with serum proteins and even red blood cell surfaces to form aggregates to adhere to tissue surfaces to cause acute cell damage [27, 28]. The inorganic nanomaterials used in the siRNA transport mostly include CaP nanoparticles [29–31], calcium carbonate [32, 33], gold nanoparticles [34–37], iron oxide [38, 39], carbon nanotubes [40, 41]. However, the challenge to decrease the levels of cytotoxicity to a clinically viable standard is still concerned.

Calcium phosphate (CaP) was developed for the delivery of DNA nearly 40 years ago [42, 43] and siRNA in recent years [44]. As a similar element of bone and teeth, CaP shows negligible cytotoxicity for siRNA delivery due to its inherent biocompatibility and biodegradability [45, 46]. Besides, CaP can be internalized into cells via the endocytosis route and the dissolution of CaP in the acidic endosome, which helps siRNA release into the cytosol and silence specific genes. Although the use of CaP for siRNA has made a lot of progress, the application of CaP-siRNA in clinical therapy is far from satisfaction, mostly because of its physical instability and weak electropositivity. CaP can be rendered with suitable properties as an excellent siRNA carrier through different modification [47]. In this review, we will focus on the recent progress of CaP nanoparticles for siRNA delivery, which are classified into poly (ethylene glycol) (PEG) coated [48–50], lipid coated [51, 52], PEI [53, 54] modified and other various polymer types.

## CaP Nanoparticles

Different polymorph of CaP, including hydroxyapatite (HAp), octacalcium phosphate (OCP) and amorphous calcium phosphate (ACP) can be synthesized by different regulation [55]. Zhao *et al.* [56] successfully prepared sericin regulated HAp and ACP in water. Moreover, bioinspired nano–micro structured-OCP was synthesized through mild electrochemical deposition method, which can be applied to hard tissue engineering [57].

As a siRNA carrier, CaP is able to target cancer cells accurately, condense the siRNA and release siRNA efficiently [48]. However, the main problems associated with CaP are its physical instability and low transfection efficiency that limit its therapeutic application [58, 59]. There is potential for further efforts to improve the stability and transfection efficiency by developing a new synthesis approach or combining CaP with other materials through different methods [60]. Therefore, different strategies have been evaluated to stabilize CaP particles and enhance transfection efficiency.

## PEG-coated

**Figure 1.**
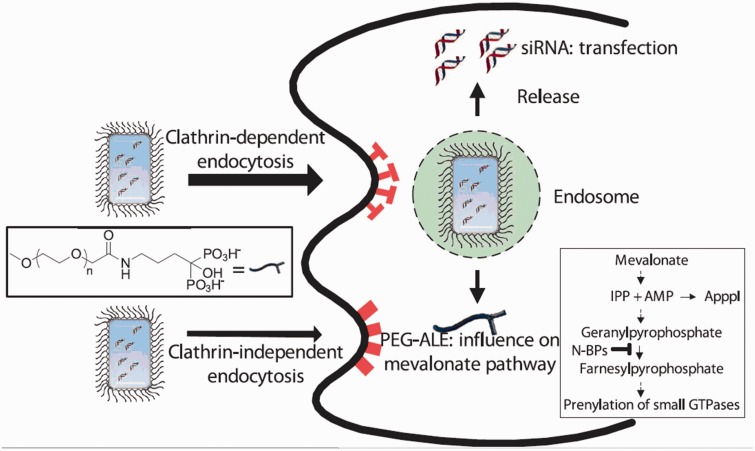
The CaP NPs coated with PEG-ALE. (Reprinted with permission from reference [50].)

PEG is used to modify CaP nanoparticles mainly because its hydrophilic and neutral features. It can help enhance the stability of compound, reduced protein adsorption and immunogenicity [61, 62]. Giger *et al.* [50] used PEGylated chelating agents (PEG-alendronate and PEG-inositol-pentakisphosphate) to prepare CaP nanoparticles through co-precipitation and found that CaP had stable enough properties to efficiently deliver siRNA *in vitro* (Fig. 1)[]. Moreover, the integration of PEG-block siRNA into CaP led to size-controllable hybrid nanoparticles, which facilitated the internalization of siRNA by cells [63]. PEG-functionalized bisphosphonate (PEG-bp) was used to prepare bp-stabilized CaP nanoparticles with the size of ∼200 nm for gene delivery [64]. PEG-bp-CaP showed effective and sustained transfection ability to cells *in vitro* with low toxicity. Giger *et al.* [50] further used PEG-alendronate (PEG-ALE) to form PEG-ALE-CaP nanoparticles for siRNA delivery. PEG-ALE-CaP-siRNA exhibited a strong silencing effect *in vitro* at both the mRNA and protein levels. The cellular trafficking study showed that PEG-ALE-CaP-siRNA internalized into cells relied largely on the clathrin-dependent endocytosis. In addition, Giger *et al.* [50] obtained the similar conclusion that CaP nanoparticles stabilized with PEGylated chelators were mainly taken up into human prostatic carcinoma (PC-3) cell by clathrin-dependent endocytosis. Zhang *et al.* [65] utilized electrostatic interactions between the anionic charges of siRNA and the cationic charges on the PEGylated CaP crystal surface to prepare CaP/siRNA nanoparticles with sizes between 90 and 200 nm, resulting in the nanoparticles showing significant gene silencing efficiency on cultured cells.

The growth of CaP can be inhibited after introducing PEG on the surface of CaP crystal. Kataoka’s [66] group has investigated the hybrid polymer CaP nanoparticles for gene delivery for one decade. First, they used PEG-block-poly (aspartic acid) (PEG-PAA) to prevent precipitation of CaP crystals. Between the CaP shore and PEG-PAA shell siRNA was surrounded by electrostatic attraction, and the core-shell nanoparticles presented efficient transfection [29]. They also verified that CaP nanoparticles are taken up by the cells through an energy-dependent endocytotic pathway. On further research, they found it was hard to increase the siRNA entrapment with a small size of particles, because siRNA and PEG-b-polyanion were competitively bound to the CaP crystal [67]. To overcome the challenge, they firstly integrated siRNA into block copolymers via a disulfide bond to form PEG-SS-siRNA conjugates [63], and demonstrated that the conjugate of siRNA with PEG via a disulfide linkage regulated the crystal growth of CaP and yielded a monodispersed nanocomposite. The prepared PEG-SS-siRNA/CaP exhibited prolonged stability in serum containing medium and substantial RNAi efficacy.

**Figure 2.**
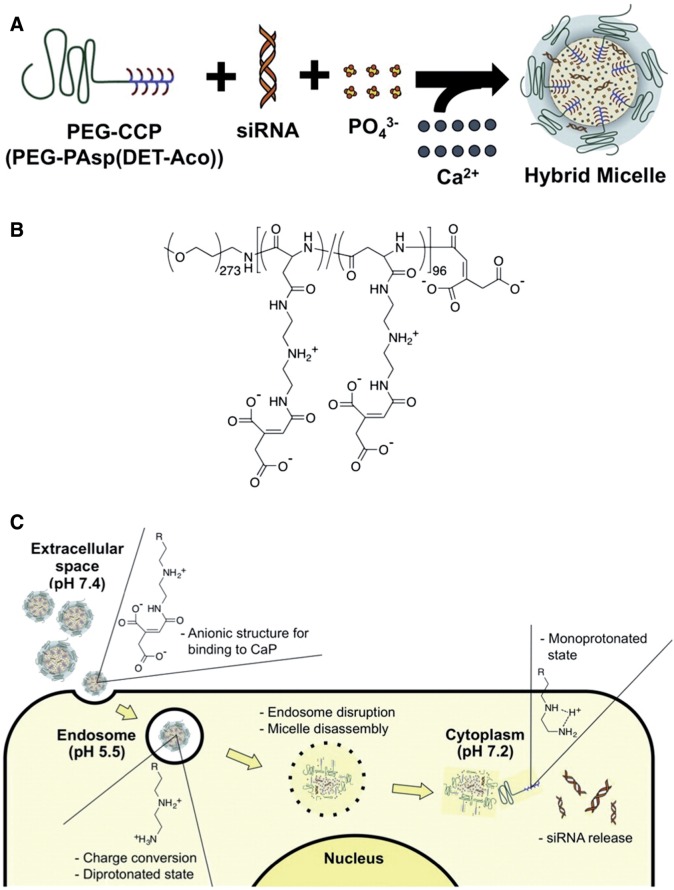
The preparation of hybrid micelles with PEG-CCP, siRNA, and CaP **(A)**. Chemical structure of PEG-PAsp (DET-Aco), termed PEG-CCP **(B)**. The cellular delivery of siRNA by PEG-CCP/CaP hybridmicelles **(C).** (Reprinted with permission from ref. [30].)

The charge-conversional polymer (CCP), of which anionic functional groups could be converted to cationic groups in an endosomal acidic condition for facilitated endosomal escape. It shows high stability at neutral and basic pHs but it becomes cleavable at acidic pH to release the cargo [68]. Kataoka’s group presented a hybrid nanocarrier system composed of CaP (comprising the block copolymer of PEG) and CCP (as a siRNA vehicle). The CaP in these nanoparticles formed a stable core to incorporate siRNA and PEG–CCP. The synthesized PEG–CCP is a non-toxic endosomal escaping unit, which induces endosomal membrane destabilization, and rapid escape of siRNA. The nanocarrier possess excellent siRNA-loading efficiency (∼80% of dose), and efficiently induced vascular endothelial growth factor (VEGF) mRNA knockdown (∼80%) in pancreatic cancer cells (Panc-1) [69]. Pittella *et al.* [70] further prepared PEG-CCP/siRNA/CaP hybrid nanoparticles for systemic delivery of siRNA to solid tumors. They found that the nanoparticles showed high gene silencing efficiency in cultured pancreatic cancer cells (BxPC3) *in vitro* and significant reduction in the subcutaneous BxPC3 tumor growth, very consistent with the significant VEGF gene silencing (∼68%) in the tumor. To further evaluate the clinical application of siRNA in cancer therapy, they developed safe and efficient nanocarrier PEG-CCP/CaP hybrid micelles to systemically deliver siRNA and studied the efficacy of PEG-CCP/siRNA/CaP in spontaneous bioluminescent pancreatic tumors from transgenic mice (Fig. 2). They found that intravenous injection of PEG-CCP/CaP-siRNA significantly reduced the luciferase-based luminescent signal from the spontaneous pancreatic tumors with no significant changes in hematological parameters in mice.

Improving the transfection efficiency of nanoparticles-based medicine in clinical therapy has been limited by the low effectiveness of nanoparticles across the cell membrane. Endocytosis has been reported to play a key role in the internalization of CaP [71]. For example, a nanoparticle coated with PEG-ALE was found to use clathrin-dependent endocytosis [50]. Tobin *et al.* [72] used PEGylated triple-shell CaP nanoparticles to absorb therapeutic siRNA and ultra-low levels of doxorubicin, and found that the nanoparticles could preferentially localize to tumor *in vivo*, be internalized by tumor cells in an increasing capacity, effectively deliver siRNA to cause significant decrease of XIAP and inhibit the tumor growth effectively. Moreover, a critical ability of the nanoparticles was to induce the apoptosis of cancer cells while avoiding normal tissue.

As promising PEGylated chelators, bps or inositol pentakisphosphate represent stabilizing agents for calcium phosphate nanoparticles. Phospholipid-methoxy PEG (PS-mPEG), whose main composition is phosphatidylserine-a main component of cell membrane, has good biocompatibility and is easy to adhere to the cell membrane. Wang *et al.* [73] reported siRNA-loaded PS-mPEG/CaP nanospheres that had good entrapment efficiency of siRNA of up to 92.86%. The nanospheres could carry siRNA into the cells then induce cell apoptosis effectively *in vitro* and carry siRNA into the tumor tissue effectively *in vivo*.

## Liposome-coated

Several research groups have successfully used lipids to inhibit the rapid growth of CaP particles for gene delivery with negligible toxicity. Though cationic liposomes are widely used transfection vectors, due to their notable efficiency [74], the cytotoxicity of liposomes makes it difficult in clinical cases [75, 76]. Khatri *et al.* [44] used liposomes composed of a neutral lipid Dipalmitoyl-sn-glycero-3-phosphocholine, a fusogenic lipid dioleoyl-sn-glycero-3-phosphoethanolamine, a PEGylated lipid (DSPE-mPEG 2000) and cholesterol to entrap CaP and siRNA. The mixtures were further grafted with cRGD to achieve targeting potential for cancer cells. The final nanoparticles had a size below 150 nm, around 80% of siRNA entrapped and higher transfection efficiency than Lipofectamine 2000. Developed liposomal-CaP showed effective protection of siRNA against serum nucleases, excellent stability against electrolyte induced flocculation, and effective target gene silence up to 24.1 ± 3.4%.

**Figure 3.**
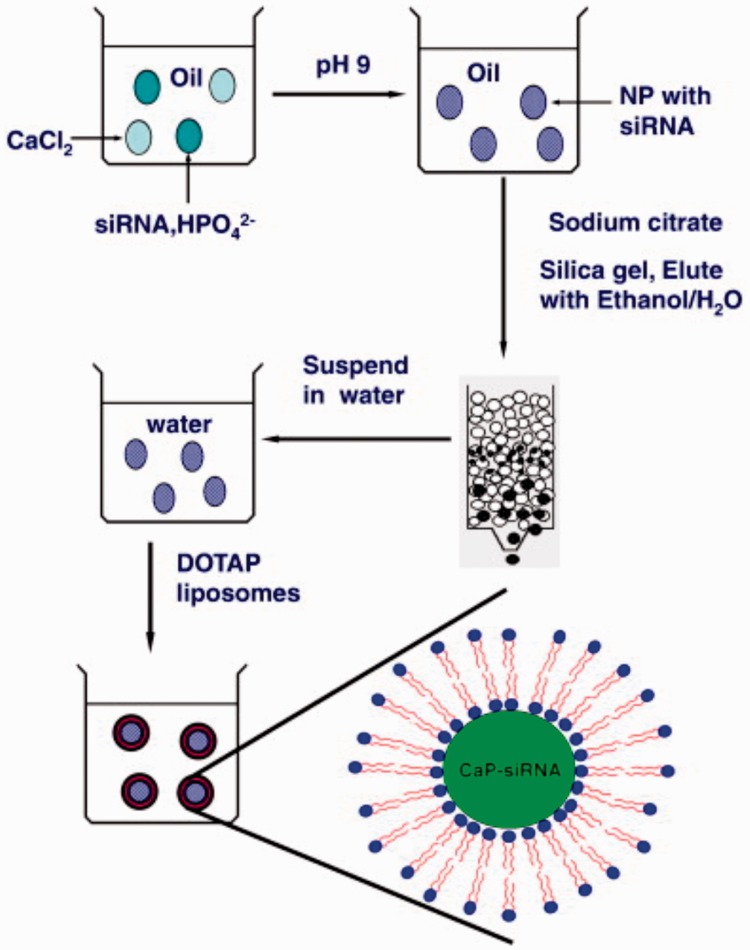
The schematic illustration of the process for LCP nanoparticles. (Reprinted with permission from reference [77].)

As Figure 3 shows, Huang and his group undertook an in-depth study on CaP modified with liposome for siRNA delivery for many years. At the beginning, they developed lipid-coated calcium phosphate (LCP) nanoparticles, which they called LCP-I afterwards, for efficient delivery of siRNA to a xenograft tumor model by intravenous administration [77]. On the previous formulation, they designed a core/shell nanoparticle structure which was named liposome-polycation-DNA (LPD) [78]. LPD showed a striking success in delivering siRNA, but the release of siRNA into the cytoplasm still needed improvement. They replaced the core of LPD with the acid-sensitive CaP and prepared LCP-I using water-in-oil microemulsions in which siRNA was entrapped. After further modifying LCP-I with a PEG linker, they found that the LCP nanoparticles had excellent siRNA delivery activity *in vitro* and in a xenograft tumor model. To improve the targeted delivery of LCP-I, they grafted the nanoparticles with PEG and anisamide (AA) ligand on the surface [79]. The improved LCP-I exhibited a 40 nm particle size and 91% siRNA encapsulation efficiency. Further therapeutic experiments revealed, siRNA formulated in LCP-I significantly increased oncogenes silencing and reduced lung metastases (similar to 70–80%). By studying the acid sensibility of LCP-I and imaging of intracellular calcium release, they found a new cargo release mechanism from the endosome that differs from the well reported proton sponge effect [80] and ion-pair effect [81]. The CaP core was dissolved at low pH, allowing dissolved calcium and phosphate ions to increase the osmotic pressure and resulted in the swelling of the endosome, following this, the endosome burst releasing the siRNA cargo into cytoplasm [77].

**Figure 4.**
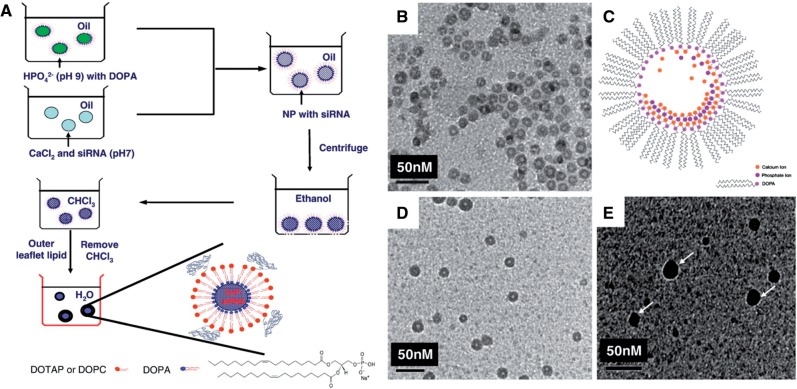
The schematic illustration for the preparation of LCP-II NP and the structure of DOPA **(A)**. TEM image of CaP cores coated with DOPA **(B)**. Hypothesis of the CaP core growth **(C)**. TEM images of LCP-II NPs coated with DOTAP and DSPE–PEG without **(D)** and with **(E)** negative staining. Arrows in (E) show lipid bilayer surrounding the CaP core. (Reprinted with permission from references [52].)

Dioleoylphosphatydic acid (DOPA), an anionic lipid, was employed as the inner leaflet lipid to coat the CaP cores, which entraps the siRNA. As Figure 4 shows, researchers further developed new nanoparticles, named LCP-II [52]. The outer leaflet lipid used a suitable neutral or cationic lipid to form an asymmetric lipid bilayer structure and was covered by a PEG-phospholipid conjugate. The final nanoparticles were named LCP-II with a diameter of 25–40 nm and a hollow core (Fig. 4). LCP-II could release more siRNA to the cytoplasm than LPD formulation, leading to a significant (∼40-fold *in vitro* and ∼4-fold *in vivo*) improvement in siRNA delivery.

To improve the process of LCP for combinational delivery of associated drugs, a biodegradable and amorphous core of CaP was used to encapsulate the therapeutic agents. The CaP core was coated with an asymmetrical lipid bilayer: the inner leaflet consisted of DOPA the outer leaflet was a cationic lipid DOTAP and a helper lipid cholesterol [82]. The nanoparticles were further modified with PEG phospholipid (DSPE-PEG) and AA grafted onto the surface. The LCP nanoparticles could co-deliver the chemotherapeutic drug gemcitabine monophosphate (GMP) and siRNA specific to the undruggable cMyc oncogene (cMyc siRNA) into the cytoplasm and effectively induce the apoptosis of tumor cells and the anti-tumor activity in both subcutaneous and orthotopic models of aggressive non-small-cell lung cancer over either cMyc siRNA or GMP therapy alone. In their latest research, they produced a new optimized LCP NP delivery system which could improve siRNA in cellular accumulation. Compared with Oligofectamine, a commercial RNA transfection reagent, it significantly inhibited the growth of human breast cancer cells *in vitro* [83].

## PEI modified

**Figure 5.**
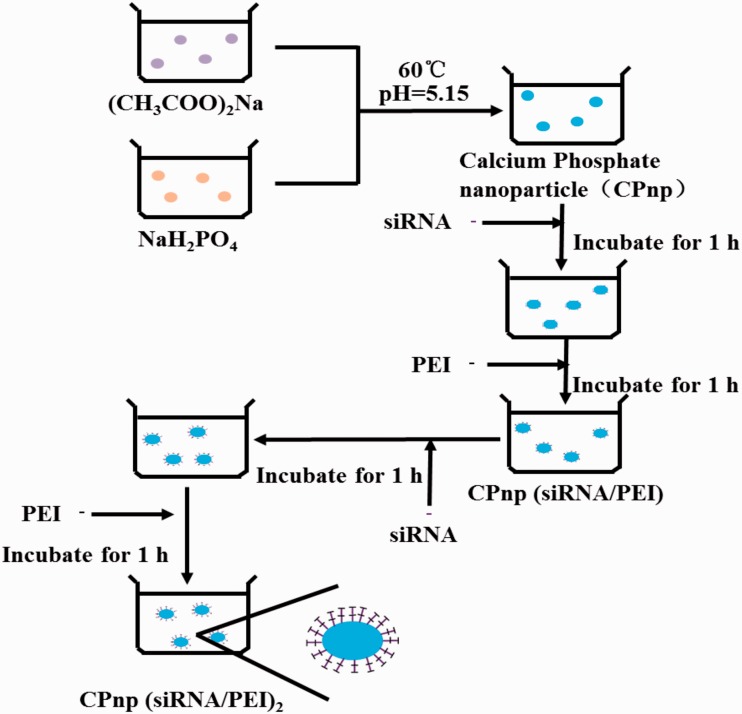
The schematic illustration of the process for CPnp (siRNA/PEI)_2_.

PEI is well known as a transfection reagent, but its cytotoxicity limits its further clinical application for the delivery of gene or drug treatment [84–86]. Devarasu *et al.* [87] described hybrid CaP nanoparticles, built by alternate deposition of siRNA and modified with PEI (CPnp(siRNA/PEI)_2_) (Fig. 5). The CaP nanoparticles showed an efficient gene silencing effect reached up to 95% in a luciferase expressing cells *in vitro* and a tumor xenograft mouse model *in vivo*. Interestingly, they found the grafting of a sugar moiety on PEI could modify the *in vivo* biodistribution of the particles without modifying the size, stability and *in vitro* efficiency.

## Other polymer coated

Besides the PEG and liposome coating mentioned above, Lee *et al.* [88] used DOPA (3, 4-dihydroxy-l-phenylalanine) modified chitosan to prevent further growth of CaP crystal by adsorption on to the crystal surface. Due to this, the prepared CaP/siRNA/DOPA-chitosan significantly increased the serum stability of siRNA, showing high cellular uptake efficiency, and had notable silencing effect of special gene. In addition, Choi *et al.* [89] developed a stable CaP nanocarrier system which could enhance intracellular uptake vastly by adding the highly cationic, glutamine-conjugated oligochitosan (Gln-OChi). CaP/siRNA//Gln-OChi was prepared to target noggin, a bone morphogenetic protein antagonist, and the osteogenic bioactivity of CaP/siRNA//Gln-OChi was further confirmed in 3D environments. Kozlova *et al.* [90] coated CaP with a shell of silica and covalently functionalized by silanization for covalent attachment of molecules like dyes or antibodies allowing siRNA to be incorporated into the cavity between the CaP surface and the outer silica shell. Then the cellular uptake of CaP nanoparticles was demonstrated on HeLa and MG-63 cell lines, and they found that the functionalization of CaP with a dendritic cell-specific antibody (CD11c) led to a cell-specific targeting *in vivo*.

To improve the diagnostic efficacy of the cargo molecules, Singh *et al.* [91] reported a biocompatible CaP-based delivery system with in situ imaging capacity. The CaP nanoparticles (formed as nanorods, 40 × 10 nm), prepared by a citrate-involved sol-gel process, showed a strong blue emission at 427 nm under fluorescence microscopy, and had excellent cell viability (over 90% for both osteoblasts and osteoclasts). The siRNA-loaded CaP exhibited sustainable siRNA release over 5 days, excellent osteoblastic uptake (96% efficiency) and gene-silencing effect, while preserving intracellular fluorescence signals. Qin *et al.* [92] prepared Pluronic F127/CaP hybrid nanoparticles (F127/CaP) (120–210 nm in diameter) by a facile room temperature method and employed them as carriers to deliver siRNA to silence tumor cell. F127/CaP had effective siRNA encapsulating efficiency up to 91.5 wt % with a loading content of 6.5 wt. %, and exhibited higher gene inhibition efficiency than traditional CaP transfection method.

## Conclusion

A wide variety of CaP nanoparticles have been developed for efficient siRNA delivery by coating suitable PEG, liposome or other polymer to CaP crystals. Generally, PEG-coated CaP is more stable than other modifications due to the colloidal stability of PEG, while liposome-coated CaP has better a transfection efficiency relying upon the cationic characteristics of the lipids. We can compare coating CaP with other modifications, such as chitosan or PEI, which provide a broader range of applications in different fields of siRNA delivery. To further improve the capability of CaP for siRNA, combinations of different coatings such as PEG and PEI have gained more attention and rapid development. Hopefully, this can lead to improvements in the inadequacy of CaP-siRNA *in vitro* and *in vivo* and provide a novel carrier system to target specific diseases, especially cancer.
